# Evidence of High Genetic Diversity and Differences in the Population Diversity of the Eucalyptus Leaf Blight Pathogen *Calonectria pseudoreteaudii* from Diseased Leaves and Soil in a Plantation in Guangxi, China

**DOI:** 10.3390/microorganisms11112785

**Published:** 2023-11-16

**Authors:** Wenxia Wu, Wenwen Li, Feifei Liu, Shuaifei Chen

**Affiliations:** 1 Research Institute of Fast-Growing Trees (RIFT), Chinese Academy of Forestry (CAF), Zhanjiang 524022, China; wuwenxia_hainan@126.com (W.W.); liww9208@126.com (W.L.); shelleyfei@163.com (F.L.); 2 College of Forestry, Nanjing Forestry University (NJFU), Nanjing 210037, China

**Keywords:** *Calonectria* leaf blight, forest disease, fungal pathogen, genetic diversity, plantation disease, population biology

## Abstract

*Calonectria pseudoreteaudii* is an important causal agent of *Eucalyptus* leaf blight in southern China. This pathogen causes *Eucalyptus* tree disease across numerous regions in southern China. In addition to diseased leaves, *C. pseudoreteaudii* has occasionally been isolated from soil in *Eucalyptus* plantations. The aim of this study was to clarify whether *C. pseudoreteaudii* causing *Eucalyptus* leaf blight in China is mainly clonally reproduced and to determine the potential spreading mechanism of *C. pseudoreteaudii* between diseased leaves and soil. To this end, 10 polymorphic microsatellite markers were analyzed to detect the genetic diversity of 97 *C. pseudoreteaudii* isolates from diseased leaves and soil in a *Eucalyptus* plantation in Guangxi Zhuang Autonomous Region, southern China. The analysis showed that the genetic diversity of the isolates from both the diseased leaves and soil was high. However, the gene and genotype diversity of the *C. pseudoreteaudii* isolates from diseased leaves were higher than those of the isolates from the soil. Moreover, all genotypes detected in the isolates from the soil were also found in the isolates from the diseased leaves. Structural analyses did not show clear population structures related to the population substrates of the diseased leaves or soil, and molecular variance analyses indicated that no significant genetic differentiation existed between the diseased leaf and soil populations. These results suggest that *C. pseudoreteaudii* in soil spreads from diseased leaves, and that an asexual cycle is the primary reproductive mode in both diseased leaf and soil populations. This is the first study on the genetic diversity and population structure of *C. pseudoreteaudii*. The high genetic diversity and spread pathways of this pathogen may pose challenges in controlling the disease. *C. pseudoreteaudii* from both diseased leaves and soils in *Eucalyptus* plantations needs to be carefully monitored for disease control and management.

## 1. Introduction

Leaf blight caused by *Calonectria* spp. is one of the most prominent diseases of *Eucalyptus* trees in Southeast Asia and South America [1,2,3,4,5,6,7,8]. *C. pseudoreteaudii* is one of the dominant species isolated from blighted *Eucalyptus* leaves, especially among some genotypes of *E. urophylla* × *E. grandis* and *E. urophylla* × *E. tereticornis* in plantations and nurseries in southern China [4,5,8,9,10]. It is a heterothallic species that resides in the *C. reteaudii* species complex [6,10,11,12,13]. In China, *C. pseudoreteaudii* has been recovered from *Eucalyptus* in Fujian, Guangdong, Guangxi, and Hainan provinces [4,5,6,8,9,10,14]. *C. pseudoreteaudii* mainly infects *Eucalyptus* trees that are less than two years old in the high temperature and humidity season. In some regions of Guangdong, Guangxi, and Hainan provinces, *C. pseudoreteaudii* has caused severe disease and nearly all leaves of infected *Eucalyptus* trees have blighted and dropped [5,8,10]. In addition to *Eucalyptus* in China, *C. pseudoreteaudii* has been isolated from *Eucalyptus* hybrids in Vietnam, India, and Indonesia; *Macadamia* sp. in Vietnam, Lao, and China; and blueberry in China [13,15,16,17,18,19,20].

Disease symptoms caused by *C. pseudoreteaudii* in *Eucalyptus* plantations include leaf and shoot blight [4,6,8,9,10,12,13,14,21]. In the early stages, the pathogen mainly infects the lower and middle leaves of *Eucalyptus* trees, resulting in grayish, water-soaked spots. Subsequently, these spots spread rapidly, becoming extensive necrotic areas, resulting in stem and shoot rot and leaf blight [8,10]. In nurseries, *C. pseudoreteaudii* causes *Eucalyptus* seedling stem, cutting, and leaf rot [8,12,14,21]. Under high-temperature and -humidity conditions, white conidiophore masses with the typical morphological characteristics of *Calonectria* spp. are observed frequently on the leaves and shoots of *Eucalyptus* trees and seedlings [8,10].

In *Eucalyptus* plantations in China, *C. pseudoreteaudii* has mainly been isolated from diseased *Eucalyptus* tissues, and occasionally from the soil under diseased *Eucalyptus* trees [4,8]. Wang and Chen [8] performed the first systematic investigation of *Eucalyptus* leaf disease caused by *Calonectria* species on the Leizhou Peninsula of southern China. The results indicated that *C. pseudoreteaudii* (identified as *C. pentaseptata* before the taxonomic revisions by Liu et al. [6]) was widely isolated from the blighted leaves of a number of *Eucalyptus* genotypes planted in different regions, and 773 *C. pseudoreteaudii* isolates were obtained from 14 sampling sites [8]. However, *Calonectria* isolates were not isolated from the soil in *Eucalyptus* plantations in the investigation. Recently, Li et al. [4] conducted several disease surveys in regions in southern China planted with *Eucalyptus* threatened by *Calonectria* leaf blight. They found that in distant regions in southern China, *C. pseudoreteaudii* was commonly isolated from the blighted leaves of *Eucalyptus*, but seldom from the soil under the diseased *Eucalyptus* trees. The results showed that 243 *C. pseudoreteaudii* isolates were obtained from six of the nine sampled regions, whereas only four *C. pseudoreteaudii* isolates were isolated from two of the nine sampled regions [4].

In recent studies, we evaluated the genotype diversity of *C. pseudoreteaudii* based on sequences of the translation elongation factor 1-alpha (*tef1*), β-tubulin (*tub2*), calmodulin (*cmdA*), and histone H3 (*his3*) gene regions [8,10]. On the basis of a number of *Eucalyptus* genotypes in multiple geographic sites, our results indicated that the genotype diversity of *C. pseudoreteaudii* was very low [8,10]. Only two genotypes were detected among the 55 tested isolates recovered from diseased *Eucalyptus* leaves by Wang and Chen [8]. In another study by our group [10], two genotypes were identified in 66 *C. pseudoreteaudii* isolates from diseased leaves, and only one genotype was present in 53 *C. pseudoreteaudii* isolates obtained from soil. The genotypes identified in the diseased leaves included those from the soil [10].

On the basis of the results from previous studies [4,8,10], we were keen to clarify whether *C. pseudoreteaudii* that causes *Eucalyptus* leaf blight in China is mainly clonally reproduced with low genetic diversity. Furthermore, we expected to clarify the differences in the population diversity of *C. pseudoreteaudii* from diseased leaves and soil within the same *Eucalyptus* plantation, which will help us to understand the potential spread pathways of *C. pseudoreteaudii* between diseased leaves and soil.

Recently, *C. pseudoreteaudii* causing leaf blight on *Eucalyptus* trees in one plantation resulted in tree damage [10], and a number of *C. pseudoreteaudii* isolates from diseased leaves and soil under the diseased trees were obtained. The objectives of this study were to (i) investigate the genetic diversity and (ii) understand the differences in the population diversity of *C. pseudoreteaudii* isolates from diseased leaves and soil using 10 polymorphic microsatellite markers.

## 2. Materials and Methods

### 2.1. Calonectria pseudoreteaudii Isolates and DNA Extraction

**Figure 1:**
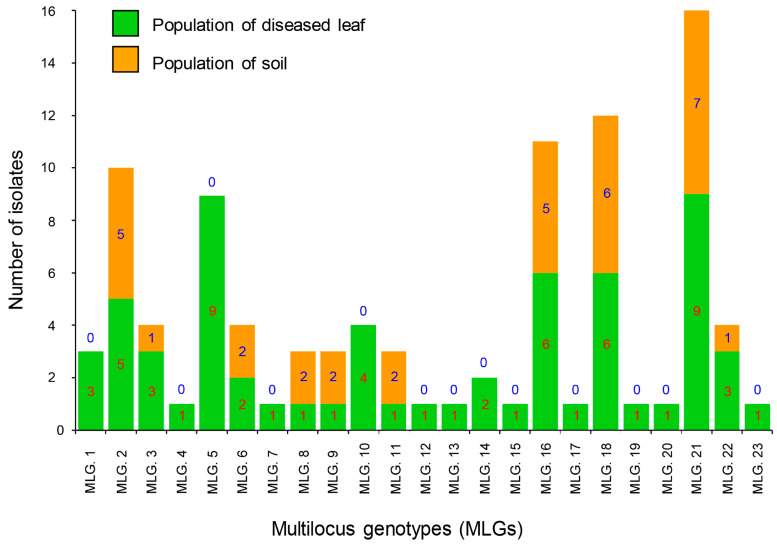
Multilocus genotypes (MLGs) generated from two *C. pseudoreteaudii* populations from diseased leaves and soil. Twenty-three genotypes were detected in the full dataset. The number of individuals residing in each genotype is indicated in each bar graph.

A total of 97 *C. pseudoreteaudii* isolates obtained from the same one-year-old *Eucalyptus* plantation in Hepu County, Beihai region, Guangxi, southern China (21°33′19.8756″ N, 109°42′27.0792″ E), in October 2018 were used in this study (Table S1). These included 94 isolates from Wu and Chen [10] and three additional isolates (CSF15861, CSF15910, and CSF15984). All 97 isolates were deposited in the culture collection (CSF) of the Research Institute of Fast-Growing Trees (RIFT), Chinese Academy of Forestry (CAF), in Zhanjiang, Guangdong Province, China. All 97 isolates were identified via DNA sequence comparisons of the *tef1*, *tub2*, *cmdA*, and *his3* gene regions ([10]; Table S1). Of the 97 isolates, 64 and 33 isolates were recovered from diseased *Eucalyptus* leaves and soil, respectively (Table S1). The 64 diseased leaf isolates were obtained from 64 *Eucalyptus* trees (one per tree). The 33 soil isolates were isolated from 18 soil samples under the diseased trees, and one or two isolates were obtained from each soil sample (Table S1). The trees in the one-year-old *Eucalyptus* plantation were five- to six-meters high, and the soil was relatively moist, as the sampling period was the rainy season in the plantation (Figure 1 in Wu and Chen [10]). The 64 isolates from the diseased leaves and 33 isolates from the soil were treated as two populations for further population studies.

To ensure that each culture represented a single individual, a single hyphal tip of each culture was moved to 2% malt extract agar (MEA) (20 g of agar powder, 20 g of malt extract powder, and 1000 µL of a 30 mg/mL streptomycin sulfate solution per liter of water) and agitated. Malt extract powder was purchased from the Beijing Shuangxuan microbial culture medium products factory, Beijing, China; agar powder was purchased from Beijing Solarbio Science & Technology Co., Ltd., Beijing, China; and the streptomycin sulfate solution was purchased from Shanghai Sango Biotech Co., Ltd., Shanghai, China, and incubated at room temperature. Mycelia were collected from 10-day-old cultures using a sterile scalpel and moved to 2 mL Eppendorf tubes for DNA extraction. Genomic DNA was extracted following the CTAB protocol [22]. In order to degrade the RNA, the extracted DNA was dissolved using 3 µL of RNase (10 mg/mL) and 30 µL of TE buffer (1 M tris-HCl and 0.5 M EDTA, pH 8.0), which were added to the extracted DNA at 37 °C for 1 h. The DNA’s concentration was measured using a NanoDrop 2000 spectrometer (Thermo Fisher Scientific, Waltham, MA, USA).

### 2.2. Microsatellite Locus Sequencing and Allele Scoring

Ten microsatellite markers with polymorphisms that were easily PCR-amplified and sequenced were used to genotype all of the *C. pseudoreteaudii* isolates [23]. The PCR reactions were performed as follows: 95 °C for 5 min, followed by 35 cycles of 95 °C for 30 s, 30 s at a 52 °C annealing temperature for each pair of primers, 72 °C for 1 min, and a final extension at 72 °C for 10 min. The PCR products were checked via agarose gel electrophoresis using a 2% agarose gel with 4S GelRed (Sangon Biotech Co., Ltd., Shanghai, China) and a 1× tris-acetate-EDTA (TAE) buffer at a constant voltage (80 V) for 40 min and viewed under UV light using a Molecular Imager Gel Doc XR system (Bio-Rad Laboratories, Inc., Hercules, CA, USA). To accurately score the alleles and confirm different alleles observed at each locus were because of the expansion or contraction of the microsatellite region instead of indels in the flanking regions, all of the amplified isolates were sequenced using Sanger sequencing. All of the PCR products were sequenced in the forward and reverse directions using corresponding primers from the Beijing Genomics Institute (Guangzhou, China). Different fragment sizes at each locus were considered to represent different alleles based on variations in the number of repeat motifs in the amplified microsatellite region. The combination of alleles from all ten genotyped markers for each isolate was regarded as a multilocus genotype (MLG). Sequences representing all alleles generated at a locus were deposited in GenBank.

### 2.3. Genotype Accumulation Curve

To test the sample number of each population and to confirm whether the number of loci used in the current study was sufficient for showing the genotypic diversity of *C. pseudoreteaudii*, the genotype accumulation curve of each population was plotted using the R package poppr [24]. The curve was assessed by randomly sampling one locus to *n* − 1 loci (*n* is the total number of loci), which counted the number of observed genotypes. The method was repeated 1000 times without a replacement to generate a distribution for each of the 10 sampled loci [24,25]. A curve reaching a plateau indicated that the loci were able to accurately represent the maximum number of MLGs present in the analyzed dataset.

### 2.4. Population Genetic Diversity Analyses

For each population from the diseased leaves and soil, GENALEX version 6.5 [26] was used to calculate the total number of alleles (Na), number of effective alleles (Nef) [27], number of MLGs, and Nei’s unbiased gene diversity (Hexp) [28]. The genotypic diversity, including the evenness (E), the number of expected MLGs for the smallest sample size based on rarefaction (eMLGs), the Shannon–Wiener index of the MLG diversity (H) [29], and Stoddart and Taylor’s index of MLG diversity (G) [30], was calculated using the R package poppr [24].

### 2.5. Population Structure, Minimum Spanning Network, and Molecular Variance Analyses

STRUCTURE 2.3.3 [31,32] was applied to assign individuals to clusters (*K*) based on their allele frequency per locus using the model-based Bayesian clustering approach. The full dataset of the 97 isolates from the diseased leaf and soil populations was submitted to STRUCTURE analyses with 20 independent iterations, with the K value ranging from 1 to 10, using the admixture model of 1,000,000 Markov Chain Monte Carlo (MCMC) iterations following a burn-in period of 250,000 iterations. The optimal number of clusters was evaluated by calculating the median values of lnPr(*K*) and Δ*K* using the online STRUCTURE HARVESTER platform [33]. The CLUMPAK (Clustering Markov Packager Across K) online platform was used to visualize the cluster pattern [34]. To determine the possible evolutionary relationships among the MLGs observed in the 97 individuals across the diseased leaf and soil populations, the poppr package was used to draw a minimum spanning network (MSN) based on Bruvo’s distance [24]. To evaluate the genetic differentiation between and within populations based on the sources (diseased leaves or soil) of the isolates, an analysis of the molecular variance [35] was conducted using GENALEX version 6.5.

### 2.6. Population Reproduction Mode

The standardized index of association (rBarD) was employed to determine whether there was random mating (rBarD close or equal to zero) or not (rBarD significantly greater than zero) in the populations. The significance of rBarD at *p* < 0.05 was evaluated by comparing the observed value of rBarD with the obtained from a total of 999 randomization samplings of the same dataset [36]. The clone-corrected datasets were used for the evaluation using the ia function in the R package poppr [24].

## 3. Results

### 3.1. Microsatellite Locus Sequencing and Allele Scoring

All 97 isolates were successfully amplified with 10 pairs of SSR markers. A total of 50 alleles were detected, with the 10 markers, ranging from 2 to 11 alleles per locus (mean 5.0). The gene diversity of the 10 loci ranged from 0.48 to 0.83, based on Nei’s indices, and the evenness of the alleles ranged from 0.66 to 0.96 (Table 1). Locus CPS156 had the highest number of observed alleles (*n* = 11), and its gene diversity was the highest (Hexp = 0.83). Locus CPS103 had the lowest number of observed alleles (*n* = 2) and the lowest gene diversity (Hexp = 0.48).

### 3.2. Genotype Accumulation Curve

The genotype accumulation curve shows that the set of nine microsatellite markers was adequate to detect 100% of the MLGs in the diseased leaf population (Supplementary Materials Figure S1). The genotype accumulation curve of the SSR data indicates that a plateau was reached with six markers in the soil population (Supplementary Materials Figure S2). Thus, 10 SSR markers were adequate to detect the genetic diversity of the isolates in each population.

### 3.3. Population Genetic Diversity Analyses

The gene diversity of the diseased leaf population (Hexp = 0.636) was higher than that of the soil population (Hexp = 0.581) (Table 2). Both the Shannon–Wiener index (H) and Stoddart and Taylor’s Index (G) show that the genotype diversity of the diseased leaf population (H = 2.795; G = 12.721) was higher than that of the soil population (H = 2.102; G = 7.118). The evenness values of the diseased leaf and soil populations were 0.80 and 0.84, respectively. The results indicate that the genotypes were relatively evenly distributed in the diseased leaf and soil populations.

**Figure 2:**
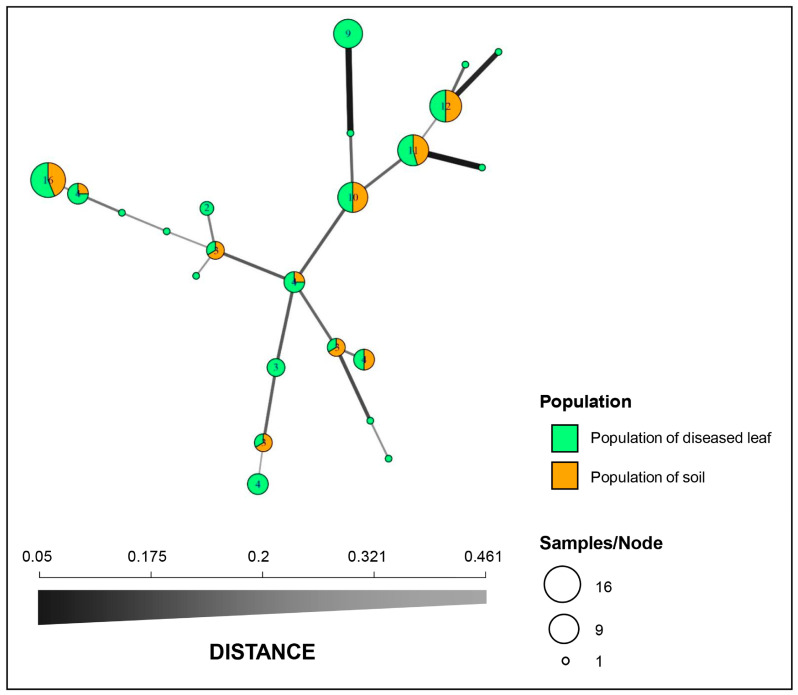
Minimum spanning network (MSN) constructed with Bruvo’s genetic distance. Each node represents a single multilocus genotype (MLG), and the node size is directly proportional to the sample size. The thickness and shading of the lines represent the genetic distance between the two genotypes (a thicker line denotes a smaller genetic distance).

A total of 23 MLGs were determined across all 97 of the *C. pseudoreteaudii* isolates. Ten genotypes were detected in the diseased leaf and soil populations. The remaining 13 genotypes were detected only in the diseased leaf population (Figure 1 and Figure 2). MLG2, MLG16, MLG18, and MLG21 were dominant genotypes in both populations.

### 3.4. Population Structure, Minimum Spanning Network, and Molecular Variance Analyses

**Figure 3:**
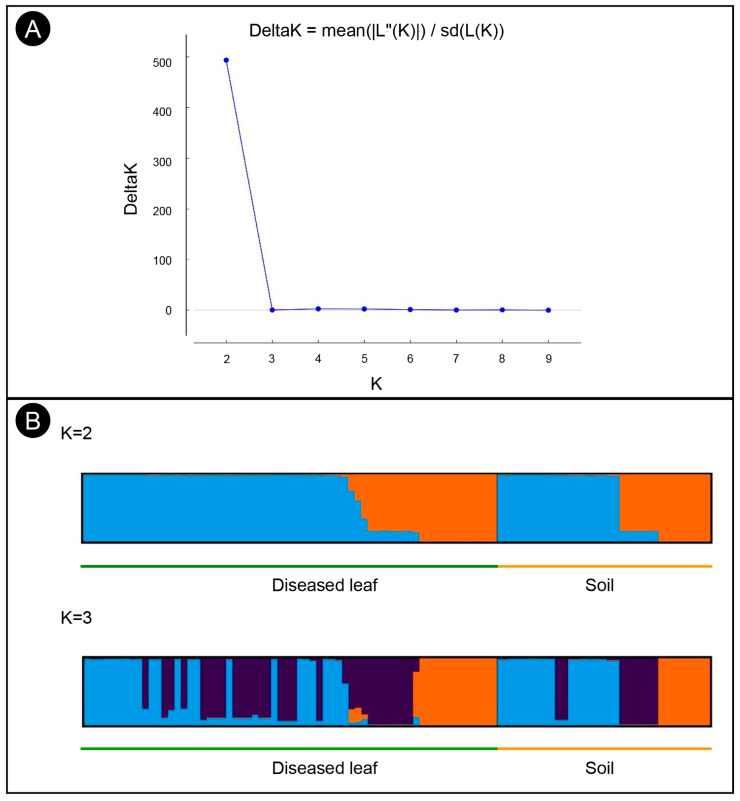
Structure analyses of *C. pseudoreteaudii* isolates from diseased leaf and soil populations. (**A**) Each individual is displayed as a bar, which is divided into K colors, where K is the possible number of clusters. (**B**) The optimal number of genetic clusters (Δ*K*) = 2.

Structure analysis of the 97 isolates suggested that K = 2 was the best number of clusters for the investigated dataset (Figure 3A). A total of 62 isolates were assigned to cluster one, which included 43 isolates from the diseased leaf population and 19 isolates from the soil population. The remaining 35 isolates were assigned to cluster two, which included 21 isolates from the diseased leaf population and 14 isolates from the soil population (Figure 3B). An admixture between clusters one and two was observed within each population, and the admixture of isolates from the diseased leaf population was more heterogeneous than that from the soil population. The minimum spanning network (MSN) analysis showed that no clear clade was specifically related to either population (Figure 2). An analysis of the molecular variance suggests that there was no significant genetic differentiation between the diseased leaf and soil populations (*p* = 0.177). A higher genetic differentiation was observed within populations (99%), and a lower variation was observed between populations (1%) (Table 3).

### 3.5. Population Reproduction Mode

The rBarD values of the diseased leaf and soil populations (population of diseased leaves: rBarD = 0.114, *p* = 0.001; population of soil: rBarD = 0.236, *p* = 0.001) were significantly greater than zero, suggesting that the asexual cycle or self-sterility represents the primary reproductive mode in diseased leaf and soil populations.

## 4. Discussion

The present study was undertaken to understand the genetic diversity and population structure of the *Eucalyptus* leaf blight pathogen *C. pseudoreteaudii* using polymorphic microsatellite markers. The genetic diversity of the isolates from diseased leaves and soil from the fungus collection site in Guangxi was high. The gene and genotype diversity of the *C. pseudoreteaudii* isolates from the diseased leaves were higher than those of the isolates from the soil. All of the genotypes detected in the soil were also found in the diseased leaves. No significant genetic differentiation existed between the diseased leaf and soil populations.

In the last decade, several studies on the population genetic diversity of *Calonectria* species have been conducted worldwide. These studies indicate the genetic diversity and population structure of the plant pathogens *C. henricotiae*, *C. pauciramosa*, and *C. pseudonaviculata* globally and of *C. pteridis* in Brazil [38,39,40,41]. Previous studies have suggested that these *Calonectria* spp. are clonally reproduced and that few genotypes are dominant in each *Calonectria* spp. [39,40,41,42]. This study indicates a high gene and genotype diversity and that multiple dominant genotypes are present in the *C. pseudoreteaudii* population at the fungus collection site in Guangxi, China. *C*. *pseudoreteaudii* in the investigated *Eucalyptus* plantation was not clonal, which increases the challenge of controlling the disease.

At the site at which *C. pseudoreteaudii* was isolated in this study, leaf blight caused by *C. pseudoreteaudii* was widely observed on the *Eucalyptus* trees throughout the plantation, whereas *C. pseudoreteaudii* was isolated only from a small proportion of the soil sampled under the diseased trees [10]. One of our aims in this study was to clarify the potential sources of *C. pseudoreteaudii* isolated from diseased leaves and soil. The genetic diversity, genetic structure, minimum spanning network, and molecular variance analyses of the diseased leaf and soil populations suggest that *C. pseudoreteaudii* in the soil was spread from the diseased leaves.

The results of previous studies consistently indicate that *C. pseudoreteaudii* is seldom isolated from soil in *Eucalyptus* plantations with leaf blight caused by this pathogen [4]. A relatively large number of *C. pseudoreteaudii* isolates were obtained from soils in a *Eucalyptus* plantation with leaf blight by Wu and Chen [10], which were collected from the *Eucalyptus* plantation a few days after rain. Some species of *Calonectria* are regarded as soil-borne fungi. These fungi can survive in soil for a long period of time because of their thick-walled microsclerotia [43]. Combining the results in this study, we hypothesized that *C. pseudoreteaudii* isolated from soil may drop from diseased leaves during the rainy season, and, therefore, these isolates are from pathogens on the diseased leaves and not soil-borne fungi. *C. pseudoreteaudii* in soil will easily die after the rainy season, especially during the dry season. It is challenging to find the appropriate season for soil sampling to obtain *C. pseudoreteaudii*. This hypothesis explains why *C. pseudoreteaudii* is seldom isolated from the soil in *Eucalyptus* plantations with leaf blight caused by this pathogen. Further research is needed to confirm this hypothesis.

The results of the standardized index of association in this study suggest that the asexual cycle or self-sterility was the primary reproductive mode in both the diseased leaf and soil populations. Previous studies have demonstrated that *C. pseudoreteaudii* is a heterothallic species [10,11]. The asexual cycle was likely the primary reproductive mode of *C. pseudoreteaudii* isolated in this study. Our previous study indicated that both mating types exist in *C. pseudoreteaudii* isolates from diseased leaves and soil [10]. The reproductive mode of *C. pseudoreteaudii* should be monitored regularly in the future. Sexual reproduction seems to drive potential recombination and could result in the appearance of more virulent genotypes of the pathogen. This will bring greater challenges to the disease control of such pathogens.

*Calonectria pseudoreteaudii* is a dominant species that causes *Calonectria* leaf blight on *Eucalyptus* trees in China. This species is widely distributed and causes disease in many *Eucalyptus*-planted regions in Fujian, Guangdong, Guangxi, and Hainan provinces [4,5,8,9,10,14,21]. Fungal populations with high levels of genetic variation have a greater evolutionary potential, which poses a greater “risk” to their hosts, and are likely to adapt more rapidly to resistant hosts than populations with low levels of genetic variation [44,45]. In this study, *C. pseudoreteaudii* was isolated from both diseased *Eucalyptus* leaves and soils, which indicates that *C. pseudoreteaudii* from both diseased leaves and soils should be considered in the disease control process. The high genetic diversity and spread of this pathogen between diseased leaves and soil may pose challenges in controlling the disease. *C. pseudoreteaudii* is common and easily obtained from diseased leaves of *Eucalyptus* plantations; however, it has been challenging to obtain *C. pseudoreteaudii* from the soil in these plantations. It is necessary to expand the sampling sites in China and neighboring countries and conduct a population biology study that will help us further clarify the genetic diversity, potential sources, and dispersal pathways of this important pathogen.

It is possible to screen for disease-tolerant *Eucalyptus* genotypes to reduce the adverse effects of leaf blight caused by *Calonectria* [8,10,46,47]. Fungal population with high levels of genetic variation are likely to have high variation in pathogenicity [48]. The results in this and previous studies suggested that diverse *C. pseudoreteaudii* isolates will be necessary for resistance screening to ensure the successful selection of *Eucalyptus* genotypes with durable disease resistance in China. Our previous research results indicated that differences in the tolerance to the tested *C. pseudoreteaudii* isolates existed among the seven tested *E. urophylla* × *E. grandis* genotypes [49]. It is necessary to test more *Eucalyptus* genetic materials to find *C. pseudoreteaudii*-tolerant *Eucalyptus* genotypes.

## 5. Conclusions

This study proved that the genetic diversity of *C. pseudoreteaudii* isolates from diseased leaves and soil from a fungus collection site in Guangxi was high, and that *C. pseudoreteaudii* in soil may spread from diseased leaves. The results in this study showed that *C. pseudoreteaudii* is an important pathogen causing *Eucalyptus* leaf blight in southern China. In the process of disease control and management of *Eucalyptus* leaf blight caused by *Calonectria*, *C. pseudoreteaudii* from both diseased leaves and soils in *Eucalyptus* plantations need to be carefully monitored. It is necessary to use diverse *C. pseudoreteaudii* isolates in the selection of disease-resistant *Eucalyptus* genotypes.

## Figures and Tables

**Table 1:** Details of the ten polymorphic microsatellite markers for genotyping the 97 *C. pseudoreteaudii* isolates in this study.

SSR Locus	Allele Sizes (Isolate and GenBank Accession Numbers)	No. of Alleles	Hexp ^a^	E.5 ^b^
CPS103	283 ^c^ (CSF16053, OQ858289); 286 (CSF16063, OQ858290)	2	0.48	0.96
CPS108	258 (CSF16008, OQ858291); 273 (CSF16063, OQ858292); 279 (CSF16209, OQ858293); 282 (CSF16066, OQ858294); 285 (CSF16053, OQ858295)	5	0.64	0.71
CPS113	295 (CSF16066, OQ858296); 298 (CSF16083, OQ858297); 301 (CSF16053, OQ858298); 307 (CSF15996, OQ858299)	4	0.65	0.94
CPS115	381 (CSF15996, OQ865454); 384 (CSF16094, OQ865455); 387 (CSF16066, OQ865456); 390 (CSF16053, OQ865457); 393 (CSF16023, OQ865458)	5	0.57	0.78
CPS118	356 (CSF15984, OQ865459); 368 (CSF16101, OQ865460); 377 (CSF16063, OQ865461); 380 (CSF16053, OQ865462); 383 (CSF16068, OQ865463); 386 (CSF16066, OQ865464)	6	0.79	0.92
CPS140	349 (CSF15959, OQ865465); 355 (CSF16053, OQ865466); 358 (CSF15996, OQ865467); 364 (CSF16072, OQ865468); 385 (CSF15886, OQ865469)	5	0.5	0.69
CPS144	353 (CSF16009, OQ865470); 359 (CSF16080, OQ865471); 362 (CSF16063, OQ865472); 365 (CSF15865, OQ865473)	4	0.55	0.77
CPS156	298 (CSF16066, OQ865474); 302 (CSF15996, OQ865475); 306 (CSF15984, OQ865476); 314 (CSF15933, OQ865477); 318 (CSF16085, OQ865478); 326 (CSF16053, OQ865479); 334 (CSF15964, OQ865480); 342 (CSF16211, OQ865481); 346 (CSF16094, OQ865482); 406 (CSF15916, OQ865483); 418 (CSF15861, OQ865484)	11	0.83	0.8
CPS159	368 (CSF16072, OQ865485); 374 (CSF15886, OQ865486); 377 (CSF16053, OQ865487); 380 (CSF16209, OQ865488)	4	0.51	0.66
CPS161	435 (CSF15888, OQ865489); 443 (CSF16053, OQ865490); 447 (CSF16068, OQ865491); 451 (CSF15984, OQ865492)	4	0.57	0.81

^a^ Hexp = Nei’s unbiased gene diversity [28]. ^b^ E.5 = evenness [37]. ^c^ Expected allele size.

**Table 2:** Genetic diversity of *C. pseudoreteaudii* populations from diseased leaves and soil.

Population	N ^a^	Na ^b^	Nef ^c^	Hexp ^d^	E ^e^	MLG ^f^	eMLG ^g^	H ^h^	G ^i^
Diseased leaf	64	50	3.090	0.636	0.800	23	16.310	2.795	12.721
Soil	33	34	2.535	0.581	0.840	10	10.000	2.102	7.118

^a^ N = number of individuals observed. ^b^ Na = number of total alleles observed. ^c^ Nef = number of effective alleles [27]. ^d^ Hexp = Nei’s unbiased gene diversity [28]. ^e^ E = evenness. ^f^ MLG = number of multilocus genotypes (MLG) observed. ^g^ eMLG = number of expected MLGs for the smallest sample size based on rarefaction. ^h^ H = Shannon–Wiener index of MLG diversity [29]. ^i^ G = Stoddart and Taylor’s index of MLG diversity [30].

**Table 3:** Analysis of molecular variance of *C. pseudoreteaudii* isolates from populations of diseased leaves and soil.

Source of Variation	Degrees of Freedom	Sum of Squares	Mean Squares	Estimate of Variance	Percentage of Total Variation (%)	PhiPT Value	*p*-Value ^a^
Between populations	1	4.537	4.537	0.033	1%	0.011	0.177
Within populations	95	293.422	3.089	3.089	99%	– ^b^	–
Total	96	297.959	N/A	3.122	100%	–	–

^a^ Levels of significance are based on 999 random permutations. ^b^ “–” represent data that are not available.

## Data Availability

Data are contained within the article and Supplementary Materials.

## Supplementary Materials

The following supporting information can be downloaded at: https://www.mdpi.com/article/10.3390/microorganisms11112785/s1, Table S1. The 97 *C. pseudoreteaudii* isolates analyzed in this study and alleles scored at each locus based on the results obtained through sequencing. Figure S1. The genotype accumulation curve of the 64 *C. pseudoreteaudii* isolates from the diseased leaves that were genotyped with the 10 SSR primers. The *x*-axis shows the number of loci; the *y*-axis represents the number of multilocus genotypes (MLGs) observed in the dataset; and the red, dashed line shows 100% of the multilocus genotypes observed in this study. Figure S2. The genotype accumulation curve of the 33 *C. pseudoreteaudii* isolates from the soil that were genotyped with the 10 SSR primers. The *x*-axis shows the number of loci; the *y*-axis represents the number of multilocus genotypes (MLGs) observed in the dataset; and the red, dashed line shows 100% of the multilocus genotypes observed in this study.

## References

[1] Alfenas R.F., Lombard L., Pereira O.L., Alfenas A.C., Crous P.W. (2015). Diversity and potential impact of *Calonectria* species in *Eucalyptus* plantations in Brazil. Stud. Mycol..

[2] Bose R., Banerjee S., Pandey A., Bhandari M.S., Barthwal S., Pandey S. (2023). *Calonectria* leaf blight of *Eucalyptus*: A global review. Ann. Appl. Biol..

[3] Li W.W., Chen S.F., Wingfield M.J., Duong T.A. (2023). *Calonectria queenslandica*: Causal agent of *Eucalyptus* leaf blight in Southern China. Plant Dis..

[4] Li W.W., Chen S.F., Wingfield M.J., Duong T.A. (2023). *Calonectria* species associated with diseased leaves and soils in southern China *Eucalyptus* plantations. Phytopathol. Res..

[5] Liang X.Y., Wang Q.C., Chen S.F. (2023). Phylogeny, morphology, distribution, and pathogenicity of seven *Calonectria* species from leaf blighted *Eucalyptus* in HaiNan Island, China. Plant Dis..

[6] Liu Q.L., Li J.Q., Wingfield M.J., Duong T.A., Wingfield B.D., Crous P.W., Chen S.F. (2020). Reconsideration of species boundaries and proposed DNA barcodes for *Calonectria*. Stud. Mycol..

[7] Pham N., Barnes I., Chen S.F., Liu F.F., Dang Q., Pham T., Lombard L., Crous P., Wingfield M.J. (2019). Ten new species of *Calonectria* from Indonesia and Vietnam. Mycologia.

[8] Wang Q.C., Chen S.F. (2020). *Calonectria pentaseptata* causes severe leaf disease on cultivated *Eucalyptus* in Leizhou Peninsula of southern China. Plant Dis..

[9] Chen Q.Z., Guo W.S., Ye X.Z., Huang X.P., Wu Y.Z. (2013). Identification of *Calonectria* associated with *Eucalyptus* leaf blight in Fujian Province. J. Fujian Coll. For..

[10] Wu W.X., Chen S.F. (2021). Species diversity, mating strategy and pathogenicity of *Calonectria* species from diseased leaves and soils in the *Eucalyptus* plantation in Southern China. J. Fungi.

[11] Li J.Q., Wingfield B.D., Wingfield M.J., Barnes I., Fourie A., Crous P.W., Chen S.F. (2020). Mating genes in *Calonectria* and evidence for a heterothallic ancestral state. Persoonia.

[12] Lombard L., Zhou X.D., Crous P.W., Wingfield B.D., Wingfield M.J. (2010). *Calonectria* species associated with cutting rot of *Eucalyptus*. Persoonia.

[13] Tarigan M., Pham N.Q., Jami F., Oliveira L.S., Saha M.A., Durán A., Wingfield M.J. (2023). *Calonectria* species diversity on *eucalypts* in Indonesia. South. For. J. For. Sci..

[14] Li J.Q., Wingfield M.J., Liu Q.L., Barnes I., Roux J., Lombard L., Crous P.W., Chen S.F. (2017). *Calonectria* species isolated from *Eucalyptus* plantations and nurseries in South China. IMA Fungus.

[15] Bose R., Banerjee S., Negi N., Pandey A., Bhandari M.S., Pandey S. (2022). Identification and pathogenicity of *Calonectria pseudoreteaudii* causing leaf blight of *Eucalyptus*––a new record for India. Physiol. Mol. Plant Pathol..

[16] Chen C., Liang X., Lin Y., Hsiang T., Xiang M.M., Zhang Y. (2023). First report of leaf spot and stem blight on blueberry (*Vaccinium corymbosum* ‘Bluerain’) caused by *Calonectria pseudoreteaudii* in China. Plant Dis..

[17] Crous P.W., Shivas R.G., Wingfield M.J., Summerell B.A., Rossman A.Y., Alves J.L., Adams G.C., Barreto R.W., Bell A., Coutinho M.L. (2012). Fungal Planet description sheets: 128–153. Persoonia.

[18] Jiang G.Z., Gao F., Yue H., He X.Y. (2020). First report of fruit spot of *Macadamia* sp. caused by *Calonectria pentaseptata* in China. Plant Dis..

[19] Jiang Z.E., Xie J., Wei J.G., Luo J., Wu Y.J., Luo J.T., Yang X.H., Yang X.B. (2020). First report of husk black spot on *Macadamia ternifolia* caused by *Calonectria pentaseptata* in China. Plant Dis..

[20] Phanthavong S., Daly A., Weir B., Lee D., Park D., Balmas V., Burgess L. (2023). First report of *Calonectria pseudoreteaudii* in Lao PDR associated with a leaf spot disease of *Macadamia integrifolia*. Australas. Plant Pathol..

[21] Lombard L., Chen S.F., Mou X., Zhou X.D., Crous P.W., Wingfield M.J. (2015). New species, hyper-diversity and potential importance of *Calonectria* spp. from *Eucalyptus* in South China. Stud. Mycol..

[22] van Burik J.A.H., Schreckhise R.W., White T.C., Bowden R.A., Myerson D. (1998). Comparison of six extraction techniques for isolation of DNA from filamentous fungi. Med. Mycol..

[23] Li W.W., Liu F.F., Chen S.F., Wingfield M.J., Duong T.A. (2023). High Genetic Diversity and Limited Regional Population Differentiation of Calonectria pseudoreteaudii Isolated from Diseased Eucalyptus Trees in Southern China.

[24] Kamvar Z.N., Tabima J.F., Grunwald N.J. (2014). Poppr: An R package for genetic analysis of populations with clonal, partially clonal, and/or sexual reproduction. PeerJ.

[25] Dowling M.E., Bryson P.K., Boatwright H.G., Wilson J.R., Fan Z., Everhart S.E., Brannen P.M., Schnabel G. (2016). Effect of fungicide applications on *Monilinia fructicola* population diversity and transposon movement. Phytopathology.

[26] Peakall R., Smouse P.E. (2006). GENALEX 6, genetic analysis in Excel. Population genetic software for teaching and research. Mol. Ecol. Notes.

[27] Nielsen R., Tarpy D.R., Reeve H.K. (2003). Estimating effective paternity number in social insects and the effective number of alleles in a population. Mol. Ecol..

[28] Nei M. (1978). Estimation of average heterozygosity and genetic distance from a small number of individuals. Genetics.

[29] Shannon C.E. (2001). A mathematical theory of communication. ACM SIGMOBILE Mob. Comput. Commun. Rev..

[30] Stoddart J.A., Taylor J.F. (1988). Genotypic diversity: Estimation and prediction in samples. Genetics.

[31] Falush D., Stephens M., Pritchard J.K. (2003). Inference of population structure using multilocus genotype data: Linked loci and correlated allele frequencies. Genetics.

[32] Pritchard J.K., Stephens M., Donnelly P. (2000). Inference of population structure using multilocus genotype data. Genetics.

[33] Earl D., von Holdt B. (2012). STRUCTURE HARVESTER: A website and program for visualizing STRUCTURE output and implementing the Evanno method. Conserv. Genet. Resour..

[34] Kopelman N.M., Mayzel J., Jakobsson M., Rosenberg N.A., Mayrose I. (2015). CLUMPAK: A program for identifying clustering modes and packaging population structure inferences across K. Mol. Ecol. Resour..

[35] Peakall R., Smouse P.E. (2012). GenAlEx 6.5, genetic analysis in Excel. Population genetic software for teaching and research–an update. Bioinformatics.

[36] Agapow P.M., Burt A. (2001). Indices of multilocus linkage disequilibrium. Mol. Ecol. Notes.

[37] Grünwald N.J., Goodwin S.B., Milgroom M.G., Fry W.E. (2003). Analysis of genotypic diversity data for populations of microorganisms. Phytopathology.

[38] Castroagudin V.L., Weiland J.E., Baysal-Gurel F., Cubeta M.A., Crouch J.A. (2020). One clonal lineage of *Calonectria pseudonaviculata* is primarily responsible for the boxwood blight epidemic in the United States. Phytopathology.

[39] Freitas R.G., Alfenas R.F., Guimarães L.M.S., Badel J.L., Alfenas A.C. (2019). Genetic diversity and aggressiveness of *Calonectria pteridis* in *Eucalyptus* spp.. Plant Pathol..

[40] LeBlanc N., Gehesquière B., Salgado-Salazar C., Heungens K., Crouch J.A. (2019). Limited genetic diversity across pathogen populations responsible for the global emergence of boxwood blight identified using SSRs. Plant Pathol..

[41] Li J.Q., Barnes I., Liu F.F., Wingfield M.J., Chen S.F. (2021). Global genetic diversity and mating type distribution of *Calonectria pauciramosa*: An important wide-host-range plant pathogen. Plant Dis..

[42] Wright L.P., Davis A.J., Wingfield B.D., Crous P.W., Brenneman T., Wingfield M.J. (2010). Population structure of *Cylindrocladium parasiticum* infecting peanuts (*Arachis hypogaea*) in Georgia, USA. Eur. J. Plant Pathol..

[43] Crous P.W. (2002). Taxonomy and Pathology of Cylindrocladium (Calonectria) and Allied Genera.

[44] McDonald B.A., McDermott J.M. (1993). Population genetics of plant pathogenic fungi. Bioscience.

[45] McDonald B.A., Linde C. (2002). Pathogen population genetics, evolutionary potential, and durable resistance. Annu. Rev. Phytopathol..

[46] Alfenas R.F., Freitas R.G., Pereira O.L., Coutinho M.M., Zarpelon T.G., Cândido T.S., Alfenas A.C. (2016). Screening of *Corymbia* and *Eucalyptus* species for resistance to *Calonectria pteridis* leaf blight. For. Pathol..

[47] Rodas C.A., Lombard L., Gryzenhout M., Slipper B., Wingfield M.J. (2005). Cylindrocladium blight of *Eucalyptus grandis* in Colombia. Australas. Plant Pathol..

[48] Bai Z., Qin Y., Cao K., Du J., Han Y., Tan Z., Wu G., Tian B., Yang Y., Yu Y. (2023). Genetic Diversity and Pathogenic Variation of the Rice False Smut Pathogen *Ustilaginoidea virens* from Different Rice Cultivars. Phytopathology.

[49] Wang Q.C., Chen S.F. (2020). The resistances of eight *Eucalyptus* genotypes in southern China to *Calonectria pentaseptata*. Eucalypt Sci. Technol..

