# Supplementary material for: Evidence of High Genetic Diversity and Differences in the Population Diversity of the Eucalyptus Leaf Blight Pathogen Calonectria pseudoreteaudii from Diseased Leaves and Soil in a Plantation in Guangxi, China
Source: Microorganisms. 2023 Nov 16;11(11):2785. doi: 10.3390/microorganisms11112785 (PMC10673236; doi:10.3390/microorganisms11112785)
Supplement: Supplementary file 1 [file microorganisms-11-02785-s001.zip › microorganisms-2699261-supplementary.pdf]

Supplementary Table S1. The 97 *C. pseudoreteaudii* isolates analyzed in this study and alleles scored at each locus based on results obtained through sequencing.

Isolate No.[a]	Sample No.	Genotype[b]	GenBank Accession No.[c]				Substrate	CPS103	CPS108	CPS113	CPS115	CPS118	CPS140	CPS144	CPS156	CPS159	CPS161
			tef1	*tub2*	*cmdA*	*his3*											
**CSF15861**	20181021-1-(1)	AAAA	**OQ858279**	**OQ858282**	**OQ858285**	**OQ858287**	leaf	283	282	295	387	377	355	362	418	368	435
CSF15865	20181021-1-(9)	AA--	MW285158	MW285398	–[d]	–	leaf	283	282	295	387	386	364	365	298	368	443
CSF15866	20181021-1-(11)	AAAA	MW285159	MW285399	MW290541	MW290611	leaf	283	285	301	390	380	355	365	326	377	443
CSF15877	20181021-1-(33)	AA--	MW285160	MW285400	–	–	leaf	286	273	298	390	383	355	362	318	377	447
CSF15879	20181021-1-(37)	AA--	MW285161	MW285401	–	–	leaf	286	273	298	390	377	355	362	346	377	447
CSF15881	20181021-1-(41)	AA--	MW285162	MW285402	–	–	leaf	286	273	298	390	377	355	362	346	377	447
CSF15882	20181021-1-(43)	AAAA	MW285163	MW285403	MW290542	MW290612	leaf	283	282	295	387	386	364	365	298	368	443
CSF15886	20181021-1-(51)	AA--	MW285164	MW285404	–	–	leaf	283	279	295	387	377	385	362	342	374	435
CSF15887	20181021-1-(53)	AA--	MW285165	MW285405	–	–	leaf	283	282	295	387	386	364	365	298	368	443
CSF15888	20181021-1-(55)	AA--	MW285166	MW285406	–	–	leaf	283	282	295	387	377	364	359	306	368	435
CSF15892	20181021-1-(63)	AA--	MW285167	MW285407	–	–	leaf	283	282	295	387	386	364	365	298	368	443
CSF15901	20181021-1-(81)	AA--	MW285168	MW285408	–	–	leaf	283	285	301	390	380	355	365	326	377	443
CSF15903	20181021-1-(85)	AAAA	MW285169	MW285409	MW290543	MW290613	leaf	286	273	298	390	383	355	362	318	377	447
CSF15906	20181021-1-(91)	AA--	MW285170	MW285410	–	–	leaf	286	273	298	390	383	355	362	318	377	447
CSF15908	20181021-1-(95)	AA--	MW285171	MW285411	–	–	leaf	286	273	298	390	383	355	362	318	377	447
**CSF15910**	20181021-1-(99)	AA--	**OQ858280**	**OQ858283**	–	–	leaf	286	273	301	384	383	355	362	346	377	447
CSF15912	20181021-1-(105)	AA--	MW285172	MW285412	–	–	leaf	286	273	298	390	377	355	362	346	377	447
CSF15913	20181021-1-(107)	AAAA	MW285173	MW285413	MW290544	MW290614	leaf	286	273	298	390	368	355	362	346	377	435
CSF15914	20181021-1-(109)	AA--	MW285174	MW285414	–	–	leaf	283	285	301	390	380	355	365	326	377	443
CSF15916	20181021-1-(113)	AA--	MW285175	MW285415	–	–	leaf	283	273	298	390	368	355	362	406	380	447
CSF15919	20181021-1-(119)	-A--	No	MW285416	–	–	leaf	286	273	301	390	368	355	365	346	377	447
CSF15922	20181021-1-(129)	AA--	MW285176	MW285417	–	–	leaf	283	273	301	390	383	355	362	298	377	447
CSF15925	20181021-1-(137)	AA--	MW285177	MW285418	–	–	leaf	283	273	301	390	383	355	362	298	377	447
CSF15927	20181021-1-(141)	AAAA	MW285178	MW285419	MW290545	MW290615	leaf	286	273	298	387	380	349	362	314	377	447
CSF15933	20181021-1-(153)	AAAA	MW285179	MW285420	MW290546	MW290616	leaf	286	273	298	387	383	349	362	314	377	447
CSF15936	20181021-1-(159)	AAAA	MW285180	MW285421	MW290547	MW290617	leaf	283	273	301	390	383	355	362	298	377	447
CSF15939	20181021-1-(165)	AA--	MW285181	MW285422	–	–	leaf	283	273	298	390	368	355	362	406	380	447
CSF15942	20181021-1-(171)	AA--	MW285182	MW285423	–	–	leaf	283	285	301	390	380	355	365	326	377	443
CSF15947	20181021-1-(181)	AA--	MW285183	MW285424	–	–	leaf	286	273	301	384	368	355	362	318	377	443
CSF15955	20181021-1-(195)	AAAA	MW285184	MW285425	MW290548	MW290618	leaf	283	273	301	390	383	355	362	298	377	447
CSF15956	20181021-1-(197)	AAAA	MW285185	MW285426	MW290549	MW290619	leaf	283	279	298	390	377	355	362	342	380	447
CSF15959	20181021-1-(205)	AAAA	MW285186	MW285427	MW290550	MW290620	leaf	286	273	298	387	380	349	362	314	377	447
CSF15964	20181021-1-(213)	AA--	MW285187	MW285428	–	–	leaf	283	285	301	390	380	355	365	334	377	443
CSF15965	20181021-1-(215)	AAAA	MW285188	MW285429	MW290551	MW290621	leaf	286	273	298	387	380	349	362	314	377	447
CSF15968	20181021-1-(221)	AAAA	MW285189	MW285430	MW290552	MW290622	leaf	286	273	298	387	380	349	362	314	377	447
CSF15972	20181021-1-(229)	AA--	MW285190	MW285432	–	–	leaf	286	273	301	384	368	355	362	318	377	443
**CSF15984**	20181021-1-(255)	AAAA	**OQ858281**	**OQ858284**	**OQ858286**	**OQ858288**	leaf	283	282	298	387	356	355	362	306	380	451
CSF15985	20181021-1-(257)	AAAA	MW285191	MW285433	MW290553	MW290623	leaf	286	273	298	387	380	349	362	314	377	447
CSF15987	20181021-1-(261)	AA--	MW285193	MW285435	–	–	leaf	286	273	298	387	380	349	362	314	377	447
CSF15991	20181021-1-(269)	AA--	MW285194	MW285436	–	–	leaf	283	282	295	387	377	364	359	306	368	435
CSF15993	20181021-1-(273)	AAAA	MW285195	MW285437	MW290554	MW290624	leaf	286	273	298	387	380	349	362	314	377	447

CSF15995	20181021-1-(277)	AAAA	MW285196	MW285438	MW290555	MW290625	leaf	283	282	295	387	386	364	365	298	368	443
CSF15996	20181021-1-(279)	AA--	MW285197	MW285439	–	–	leaf	283	273	307	381	383	358	362	302	380	447
CSF15998	20181021-1-(283)	AA--	MW285198	MW285440	–	–	leaf	286	273	298	387	380	349	362	314	377	447
CSF15999	20181021-1-(285)	AA--	MW285199	MW285441	–	–	leaf	286	273	298	387	380	349	362	314	377	447
CSF16001	20181021-1-(289)	AA--	MW285200	MW285442	–	–	leaf	286	273	301	384	383	358	362	326	377	447
CSF16007	20181021-1-(301)	AAAA	MW285201	MW285443	MW290556	MW290626	leaf	286	273	298	390	368	355	362	346	377	435
CSF16008	20181021-1-(303)	AA--	MW285202	MW285444	–	–	leaf	283	258	298	390	368	349	353	342	374	447
CSF16009	20181021-1-(305)	AA--	MW285203	MW285445	–	–	leaf	283	258	298	390	368	349	353	342	374	447
CSF16010	20181021-1-(307)	AAAA	MW285204	MW285446	MW290557	MW290627	leaf	283	282	295	387	386	364	365	298	368	443
CSF16013	20181021-1-(313)	AA--	MW285205	MW285447	–	–	leaf	283	282	295	387	377	364	359	306	368	435
CSF16016	20181021-1-(319)	AABA	MW285206	MW285448	MW290558	MW290628	leaf	283	258	298	390	368	349	353	342	374	447
CSF16017	20181021-1-(321)	AA--	MW285207	MW285449	–	–	leaf	283	285	301	390	380	355	365	326	377	443
CSF16018	20181021-1-(323)	AAAA	MW285208	MW285450	MW290559	MW290629	leaf	286	273	298	390	383	355	362	318	377	447
CSF16019	20181021-1-(325)	AA--	MW285209	MW285451	–	–	leaf	286	273	298	390	368	355	362	346	377	435
CSF16021	20181021-1-(329)	AA--	MW285210	MW285452	–	–	leaf	283	282	295	387	386	364	365	298	368	443
CSF16023	20181021-1-(333)	AAAA	MW285211	MW285453	MW290560	MW290630	leaf	283	273	301	393	383	355	362	298	377	447
CSF16024	20181021-1-(335)	AA--	MW285212	MW285454	–	–	leaf	283	258	298	390	368	349	353	342	374	447
CSF16027	20181021-1-(341)	AAAA	MW285213	MW285455	MW290561	MW290631	leaf	283	273	301	390	383	355	362	298	377	447
CSF16031	20181021-1-(349)	AAAA	MW285214	MW285456	MW290562	MW290632	leaf	283	282	295	387	386	364	365	298	368	443
CSF16035	20181021-1-(357)	AA--	MW285215	MW285457	–	–	leaf	283	285	301	390	380	355	365	326	377	443
CSF16039	20181021-1-(365)	AA--	MW285216	MW285458	–	–	leaf	283	285	295	384	383	355	365	326	377	443
CSF16042	20181021-1-(371)	AA--	MW285217	MW285459	–	–	leaf	283	273	301	390	383	355	362	298	377	447
CSF16045	20181021-1-(377)	AA--	MW285218	MW285460	–	–	leaf	283	282	295	387	386	364	365	298	368	443
CSF16053	20181021-1-(12)	AAAA	MW285225	MW285467	MW290564	MW290634	soil	283	285	301	390	380	355	365	326	377	443
CSF16054	20181021-1-(12)	AA--	MW285226	MW285468	–	–	soil	283	285	301	390	380	355	365	326	377	443
CSF16062	20181021-1-(38)	AA--	MW285232	MW285474	–	–	soil	283	285	301	390	380	355	365	326	377	443
CSF16063	20181021-1-(38)	AA--	MW285233	MW285475	–	–	soil	286	273	298	390	377	355	362	346	377	447
CSF16066	20181021-1-(44)	AAAA	MW285236	MW285478	MW290566	MW290636	soil	283	282	295	387	386	364	365	298	368	443
CSF16068	20181021-1-(44)	AA--	MW285238	MW285480	–	–	soil	283	273	301	390	383	355	362	298	377	447
CSF16072	20181021-1-(52)	AA--	MW285242	MW285484	–	–	soil	283	282	295	387	386	364	365	298	368	443
CSF16076	20181021-1-(54)	AA--	MW285246	MW285488	–	–	soil	283	282	295	387	386	364	365	298	368	443
CSF16077	20181021-1-(54)	AA--	MW285247	MW285489	–	–	soil	283	282	295	387	386	364	365	298	368	443
CSF16080	20181021-1-(56)	AA--	MW285250	MW285492	–	–	soil	283	282	295	387	377	364	359	306	368	435
CSF16083	20181021-1-(56)	AA--	MW285252	MW285494	–	–	soil	286	273	298	390	383	355	362	318	377	447
CSF16085	20181021-1-(64)	AA--	MW285254	MW285496	–	–	soil	286	273	298	390	383	355	362	318	377	447
CSF16089	20181021-1-(64)	AA--	MW285257	MW285499	–	–	soil	283	282	295	387	386	364	365	298	368	443
CSF16090	20181021-1-(86)	AAAA	MW285258	MW285500	MW290568	MW290638	soil	286	273	298	390	383	355	362	318	377	447
CSF16094	20181021-1-(106)	AA--	MW285262	MW285504	–	–	soil	286	273	301	384	383	355	362	346	377	447
CSF16095	20181021-1-(106)	AA--	MW285263	MW285505	–	–	soil	286	273	301	384	383	355	362	346	377	447
CSF16101	20181021-1-(154)	AAAA	MW285269	MW285511	MW290577	MW290647	soil	286	273	301	390	368	355	365	346	377	447
CSF16102	20181021-1-(154)	AA--	MW285270	MW285512	–	–	soil	286	273	301	390	368	355	365	346	377	447
CSF16114	20181021-1-(172)	AA--	MW285281	MW285523	–	–	soil	283	285	301	390	380	355	365	326	377	443
CSF16116	20181021-1-(196)	AAAA	MW285283	MW285525	MW290580	MW290650	soil	286	273	301	384	368	355	362	318	377	443
CSF16117	20181021-1-(196)	AA--	MW285284	MW285526	–	–	soil	286	273	301	384	368	355	362	318	377	443
CSF16134	20181021-1-(222)	AAAA	MW285301	MW285539	MW290590	MW290660	soil	286	273	298	390	383	355	362	318	377	447

CSF16135	20181021-1-(222)	AA--	MW285302	MW285540	–	–	soil	286	273	298	390	383	355	362	318	377	447
CSF16138	20181021-1-(228)	AA--	MW285305	MW285543	–	–	soil	283	273	301	390	383	355	362	298	377	447
CSF16139	20181021-1-(228)	AA--	MW285306	MW285544	–	–	soil	283	273	301	390	383	355	362	298	377	447
CSF16141	20181021-1-(230)	AA--	MW285308	MW285546	–	–	soil	283	285	301	390	380	355	365	326	377	443
CSF16142	20181021-1-(230)	AA--	MW285309	MW285547	–	–	soil	283	285	301	390	380	355	365	326	377	443
CSF16163	20181021-1-(278)	AAAA	MW285328	MW285566	MW290597	MW290667	soil	283	282	295	387	386	364	365	298	368	443
CSF16164	20181021-1-(278)	AA--	MW285329	MW285567	–	–	soil	283	282	295	387	386	364	365	298	368	443
CSF16209	20181021-1-(320)	AA-A	MW285369	MW285610	–	MW290673	soil	283	279	298	390	377	355	362	342	380	447
CSF16211	20181021-1-(320)	AAAA	MW285371	MW285612	MW290603	MW290674	soil	283	279	298	390	377	355	362	342	380	447
CSF16235	20181021-1-(342)	AAAA	MW285392	MW285632	MW290609	MW290681	soil	283	273	301	390	383	355	362	298	377	447
CSF16236	20181021-1-(342)	AA--	MW285393	MW285633	–	–	soil	283	273	301	390	383	355	362	298	377	447

[a] CSF: Culture collection located at Research Institute of Fast-growing Trees (RIFT)/China Eucalypt Research Centre (CERC), Chinese Academy of Forestry (CAF), Zhanjiang 524022, Guangdong Province, China. Isolates sequenced and identified in this study were indicated in bold. [b] Genotype within each *Calonectria* species, determined by sequences of the *tef1*, *tub2*, *cmdA*, and *his3* regions. [c] *tef1*: translation elongation factor 1-alpha; *tub2*: β-tubulin; *cmdA*: calmodulin; *his3*: histone H3. Sequences generated in this study were indicated in bold. [d] "–" represents the relative locus was not amplified in the current study.

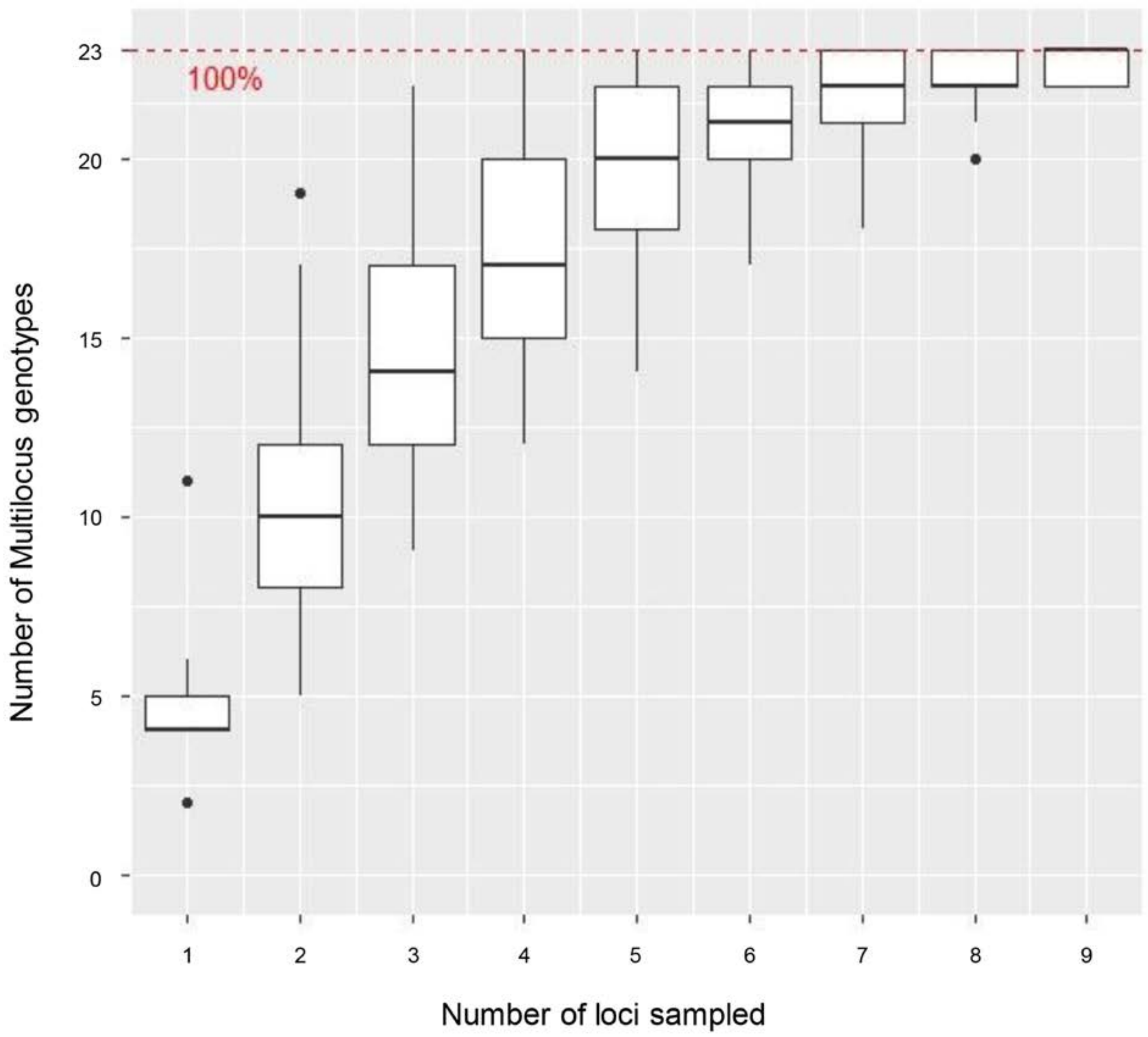

Supplementary Figure S1.

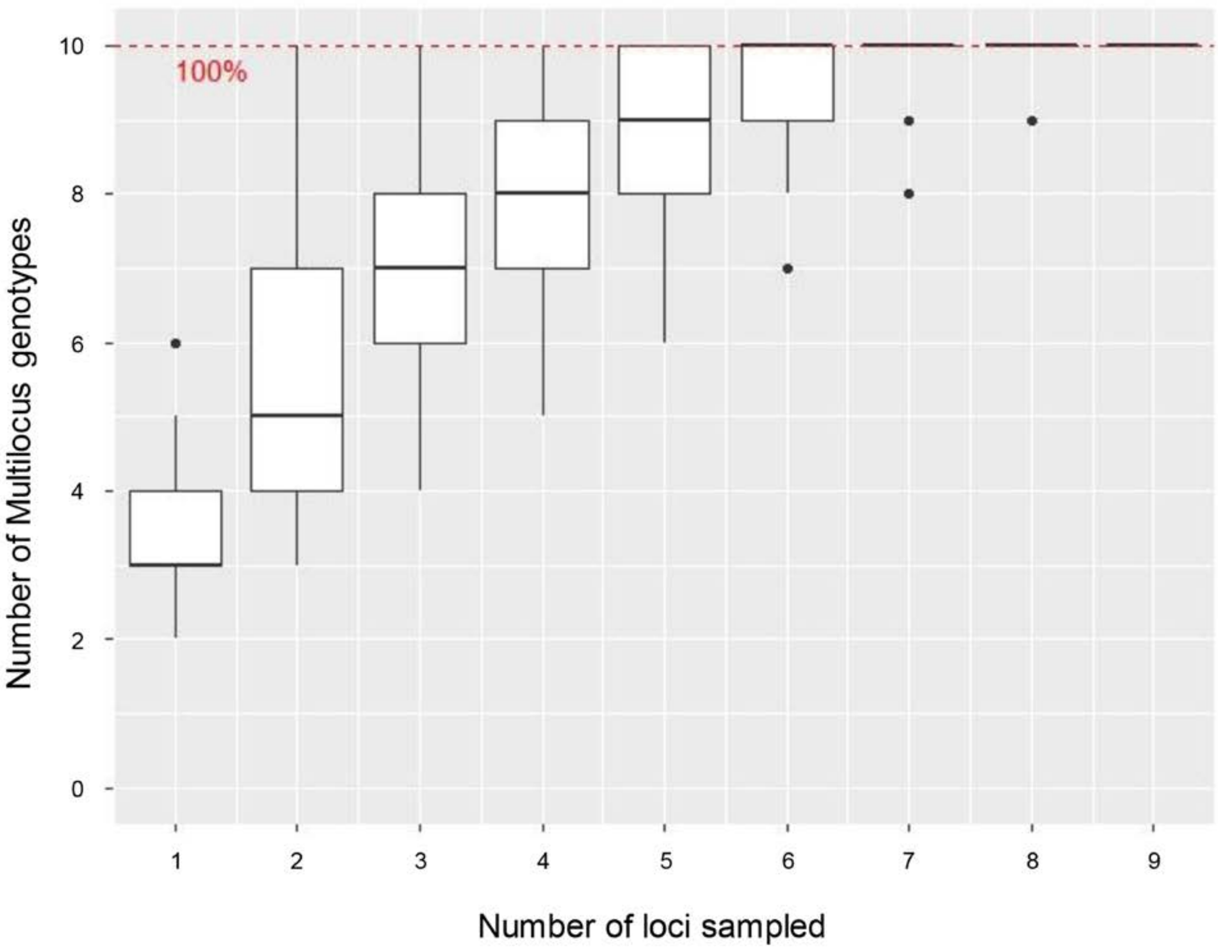

Supplementary Figure S2.
